# Health disparities in the burden of pneumococcal disease in US adults

**DOI:** 10.1186/s41479-026-00197-z

**Published:** 2026-04-07

**Authors:** Nicole Cossrow, Salini Mohanty, Kelly D. Johnson, Thomas Weiss, Sheba Nellore, Lindsay McNamee, Laura De Benedetti, Elmira Flem, Kristen Feemster

**Affiliations:** 1 Merck & Co., Inc., Rahway, NJ USA; 2 Certara USA Inc., 126 East Lincoln Ave., P.O. Box 2000, Rahway, NJ 07065 USA

**Keywords:** Pneumococcal disease, Disease burden, Health disparities, Vaccination

## Abstract

**Background:**

There are significant racial and environmental disparities in the burden of pneumococcal disease. Understanding the role of social determinants of health (SDoH) on pneumococcal disease can help health authorities identify health inequities and develop interventions to reduce these disparities. This targeted literature review (TLR) aimed to examine the clinical and economic burden of pneumococcal disease in US adults with a focus on SDoH such as race, urbanicity, and socioeconomic status.

**Methods:**

A TLR of studies published between January 2012 and July 2024 was conducted using PubMed (via Medline) and Centers for Disease Control and Prevention (CDC) surveillance data. Supplementary searches were made on Google Scholar to address data gaps. Outcomes of interest were incidence, prevalence, mortality, healthcare resource use, costs, and vaccine coverage rates by race/ethnicity, urbanicity (population density), and socioeconomic variables (income, education, employment status and home ownership).

**Results:**

Of 4,609 identified publications, 12 studies were included. Black adults had the highest incidence and mortality rates and longest hospital stay due to pneumococcal disease across all adult age groups. Additionally, Black (compared to non-Black) adults were more likely to be hospitalized at younger ages (50–64 years). Black adults ≥50 years incurred significantly higher pneumococcal disease hospitalization costs compared to non-Black adults. Lower urbanicity displayed higher mortality rates for adults with pneumonia. Adult patients 18–64 years living in more disadvantaged areas had a higher risk of hospitalization for IPD. Similarly, adults living in higher levels of area-based poverty had increased rates of CAP hospitalizations. Incidence of community-acquired pneumonia (CAP) was higher in early retirees and their adult dependents compared to their employed counterparts and adult dependents. Vaccination rates were lower in Black adults, rural residents, those with lower SES, education or income, blue-collar workers, and those who did not own a home.

**Conclusion:**

Disparities in pneumococcal disease burden and vaccination uptake exist among US adults, particularly among Black adults, rural residents and those with lower education and income. There is paucity of studies examining disparities in pneumococcal disease and inequities according to race, urbanicity, and socioeconomic status warranting further investigation of the topic to inform prevention strategies.

## Background

*Streptococcus pneumoniae* (*S. pneumoniae*) is a bacterial pathogen that causes pneumococcal disease [1]. Pneumococcal disease can range in severity from non-invasive (acute otitis media, non-bacteremic pneumonia and otitis media) to invasive disease (meningitis, bacteremia, and bacteremic pneumonia) [2]. Despite vaccination efforts, S. pneumoniae continues to be the leading cause of bacterial pneumonia globally [3]. While invasive pneumococcal disease (IPD) is less prevalent, it carries a high risk of morbidity and mortality [4]. Overall, pneumococcal disease is associated with a significant clinical and economic burden [4–7], with pneumococcal pneumonia being the most common manifestation of pneumococcal disease in adults in the United States (US) [1]. 

In addition to the clinical and economic impacts, there are significant racial and environmental disparities in the burden of pneumococcal disease. These disparities in pneumococcal disease burden are included under the social determinants of health (SDoH) framework, which Healthy People 2030 defined as “the conditions in the environments in which people are born, live, learn, work, play, worship, and age that affect a wide range of health, functioning, and quality-of-life outcomes and risks.” [8] Environmental factors such as urbanicity and socioeconomic status (SES) play a role in the distribution of pneumococcal disease in the US. Additionally, pneumococcal disease burden is characterized by marked racial disparities in the US, with higher burden observed among Black versus non-Black populations [9, 10]. 

Pneumococcal disease prevention is key, however there are clear disparities in vaccination coverage. Notably, Black adults are significantly less likely to be vaccinated than non-Black adults [10]. Considering the role and impact of the SDoH on pneumococcal disease and vaccination coverage can help health authorities identify health inequities and develop interventions aimed to reduce disparities and improve overall health and well-being. However, research into the impact that SDoH have on pneumococcal disease in the US is limited. Knowledge gaps include how the SDoH affect mortality, morbidity, resource use and associated costs, and vaccination rates. An enhanced understanding of these topics will be instrumental in understanding the unmet medical need in pneumococcal disease prevention. Therefore, the objective of this study was to conduct a targeted literature review (TLR) of the clinical and economic burden of pneumococcal disease in US adults with a focus on examining SDoH such as race/ethnicity, urbanicity (population density), and SES (income, education, employment status, poverty level, and home ownership), to help direct prevention efforts.

## Methods

### Search strategy

A targeted review of the literature published from January 2012 to July 2024 was conducted to identify original research studies reporting data on pneumococcal disease burden (incidence, prevalence, mortality), healthcare resource use, cost, and vaccine coverage rates by race/ethnicity, urbanicity (population density), and socioeconomic variables (income, education, employment status and home ownership). Since the focus of this TLR was on the burden of pneumococcal disease in the US, PubMed (via Medline) was the main database to be searched because it captures the vast majority of studies published in the US. Publications and surveillance data from the Centers for Disease Control and Prevention’s (CDC) website were also searched. The original search was supplemented with targeted searches via Google Scholar in areas for which data were not identified via Pubmed or the CDC website.

The search strategy was conducted using search terms related to health disparities in pneumococcal disease (*pneumococcal disease OR Streptococcus pneumonia OR non-bacteremic pneumococcal pneumonia OR all-cause pneumonia*) in the United States, including epidemiological outcomes *(incidence OR prevalence OR mortality)*, morbidity, healthcare resource utilization and cost (*cost OR unemployment OR productivity OR absenteeism OR hospital admission OR length of stay [LOS])*, and vaccine coverage *(vaccination OR vaccine coverage*).

### Study selection and literature screening

Study selection for inclusion was based on the Patient, Intervention, Comparison, Outcome and Time (PICOTS) framework (Table 1).

Screening of the retrieved publications was conducted by a single reviewer in two stages: title/abstract and full-text article. About 20% of citations were screened by a second reviewer to ensure consistent understanding and application of PICOTS criteria.

**Table 1:** PICOTS study inclusion criteria

	Inclusion Criteria
Population(s)	All adults 18 years and older
Interventions	NA
Comparisons	To explore health disparities on the burden of pneumococcal disease and pneumococcal vaccine coverage rates (VCRs) across the following groups/sub-groups: • Race/ethnicity • Urbanicity (population density) • Socioeconomic status: income, education, employment status, poverty level, home ownership
Outcomes	• Pneumococcal vaccine coverage rate • For all forms of pneumococcal disease (invasive pneumococcal disease (IPD), non-bacteremic pneumococcal pneumonia (NBPP), all-cause pneumonia (ACP)) with a focus on *S. pneumoniae*: – Disease incidence and prevalence – Mortality – Morbidity – Costs – Healthcare resource utilization (hospital admissions, length of stay, etc.), productivity burden (days missed from work, unemployment)
Time	2012 to July 31st, 2024
Study design	All study designs that include relevant information, except for case reports and case series
Language	English

ACP, all-cause pneumonia; IPD, invasive pneumococcal disease; NA, not applicable; NBPP; non-bacteremic pneumococcal pneumonia; *S. pneumoniae*: Streptococcus pneumonia; VCR, vaccine coverage rate

## Results

Of 4,609 publications identified, 12 (0.3%) studies were included: 5 for race and ethnicity, 1 for urbanicity, 3 for SES, 2 for both urbanicity and SES, and 1 for race and ethnicity along with urbanicity. Results are reported by pneumococcal disease manifestation (IPD, NBPP, and ACP), where available.

### Disparities by race and ethnicity

Racial and ethnic disparities in pneumococcal disease burden in US adults are well characterized and represent the factor with the most available evidence.

#### Incidence

In the US, non-White individuals experienced a higher burden of IPD compared to White individuals. According to the 2022 Active Bacterial Core surveillance (ABCs) report, the overall IPD incidence rate per 100,000 population was highest among the Black population 0–85 years old at 12.1 per 100,000, compared to the White population (7.9 per 100,000) and other races (5.7 per 100,000) [6]. A similar pattern was evident in 2017–2018 among adults ≥ 19 years living with human immunodeficiency virus (HIV) identified through the ABCs (*N* = 2440), with the IPD incidence in non-Hispanic Black adults being nearly twice that of non-Hispanic White adults [11]. Additionally, a retrospective study using state-wide surveillance data in Alaska among adults ≥ 18 years between 2011 and 2020 (*N* = 1164) found that the incidence of IPD among Alaska Native adults was significantly higher than among non-Alaska Native adults (age-adjusted rates 65/100,000 vs. 14/100,000) [12]. 

#### Mortality

**Fig. 1:**
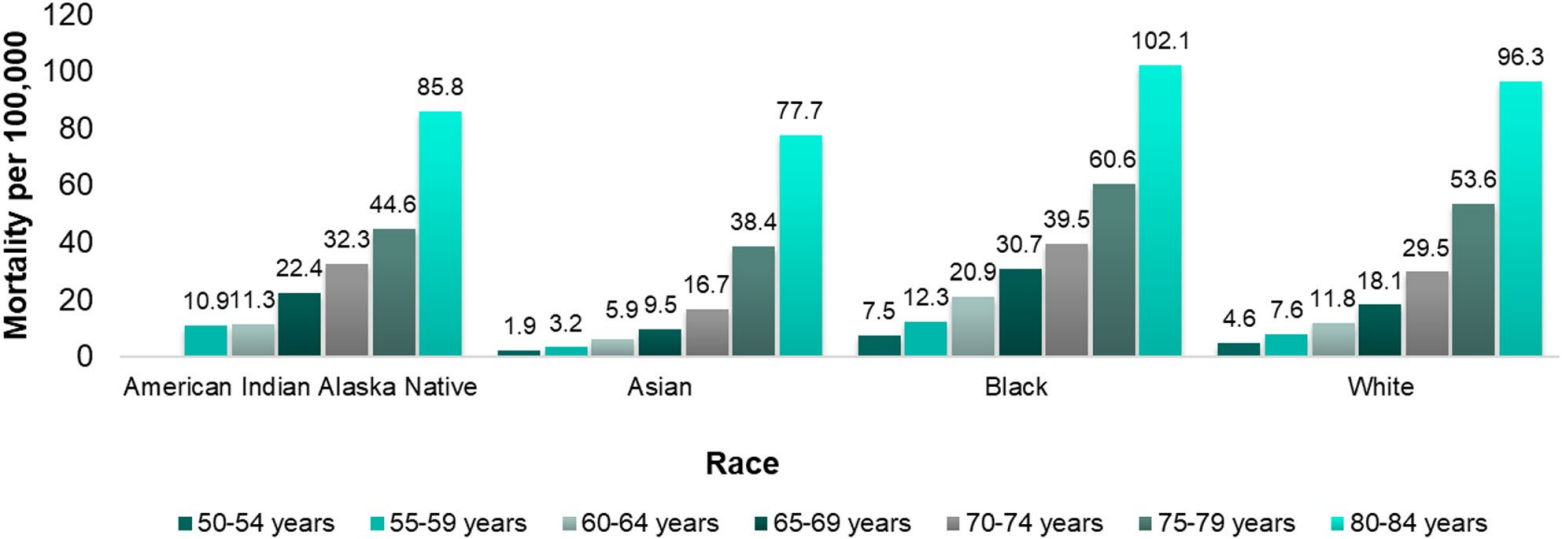
ACP* mortality rates by race and age based on CDC data (2019). *ACP, all-cause pneumonia. Source: Adapted from CDC 2021 [13]. Pre-pandemic data presented

Mortality differences reflect the same pattern observed in incidence. Studies consistently showed that older Black adults reported higher pneumococcal disease-related mortality rates compared to their non-Black counterparts. In 2019, the CDC WONDER Underlying Cause of Death database reported the highest mortality rates among Black adults ≥ 50 years old with all-cause pneumonia (ACP) [13]. This disparity persisted across age groups 50 and older, with mortality rates increasing with age for all races, but remained notably higher for Black adults (Fig. 1) [13]. A 2014 study using data from the National Health Interview Survey, National Center for Health Statistics and National Inpatient Sample database (*N* = 2,193,296), found a similar trend, with Black adults having significantly higher proportions of death due to pneumococcal disease (IPD and non-bacteremic pneumococcal pneumonia [NBPP]) during hospitalization versus non-Black adults at age 65 years (15.1% vs. 10.2%, respectively; *p* < 0.001) and 80 years (48.4% vs. 36.4%, respectively; *p* < 0.001) [10]. Across all age groups, the age-specific relative likelihood of mortality was 1.1%−8.6% higher in the Black versus non-Black population [10]. 

#### Healthcare resource utilization

Disparities were also observed in hospital care. Black adults hospitalized with pneumococcal disease (IPD and NBPP) experienced longer hospital stays compared to non-Black adults, according to an analysis of the National Health Interview Survey, National Center for Health Statistics and National Inpatient Sample database (*N* = 2,193,296) from a 2014 study (Table 1) [10]. Overall, Black adults ≥ 50 years hospitalized with pneumococcal disease had an average LOS of 9.1 days compared to 7.5 days for non-Black adults (*p* < 0.001) [10]. For hospitalization rates, Black adults were more likely to be hospitalized at younger ages, with 45.0% of hospitalizations occurring between the ages of 50 and 64 years compared to 26.9% of hospitalizations among non-Black adults [10]. Conversely, hospitalized Black adults were less likely to be in the oldest age group (≥ 80 years) compared to their non-Black counterparts (19.1% vs. 34.1%) [10]. 

#### Costs

In addition to longer hospital stays, Black adults hospitalized with pneumococcal disease (IPD and NBPP) also experienced higher costs compared to non-Black adults according to the 2014 National Health Interview Survey analysis. (Table 1) [10]. These disparities were most pronounced among older adults. For patients ≥ 50 years, including those discharged alive and those who died in the hospital, the average pneumococcal disease-related hospitalization cost was substantially higher for Black adults than for non-Black adults ($20,733 vs. $17,844; *p* < 0.001) [10] (Table 2).

**Table 2:** Racial disparities related to hospitalization costs and length of stay

	Adult Patient Group	Comparison (Black adults vs. Non-black adults)	
		IPD	NBPP
Length of Stay (LOS)	LOS of discharged adult patients	Black adults had a **significantly longer** LOS compared to non-Black adults (*p* < 0.001).	Black adults had a **significantly longer** LOS compared to non-Black adults (*p* < 0.001).
	LOS of adult patients who died in the hospital	No significant difference in LOS between Black and non-Black adults.	Black adults had a **significantly longer** LOS compared to non-Black adults (*p* < 0.001)
Average Hospitalization costs	For both adults discharged alive and adults who died in the hospital	No significant difference in costs between racial groups except in adults **≥ 80 years** where Black adults had **higher costs** (*p* = 0.01).	Black adults **≥ 65 years** incurred significantly **higher costs** compared to non-Black adults (*p* < 0.05), but in adults **aged 50–64 years**, costs were **similar across racial groups**.

IPD, invasive pneumococcal disease; LOS, length of stay; NBPP, non-bacteremic pneumococcal pneumonia

Source: Nowalk 2019 [10]

Exact cohort/sample sizes are unknown; Cohort sizes were based on US Census data on 65-year-olds in 2020; Cohorts were segmented based on the presence or absence of chronic health conditions relevant to differential pneumococcal disease risk, using age-and race-specific 2013–2014 National Health Interview Survey and CDC data as previously described

#### Vaccination rate

**Fig. 2:**
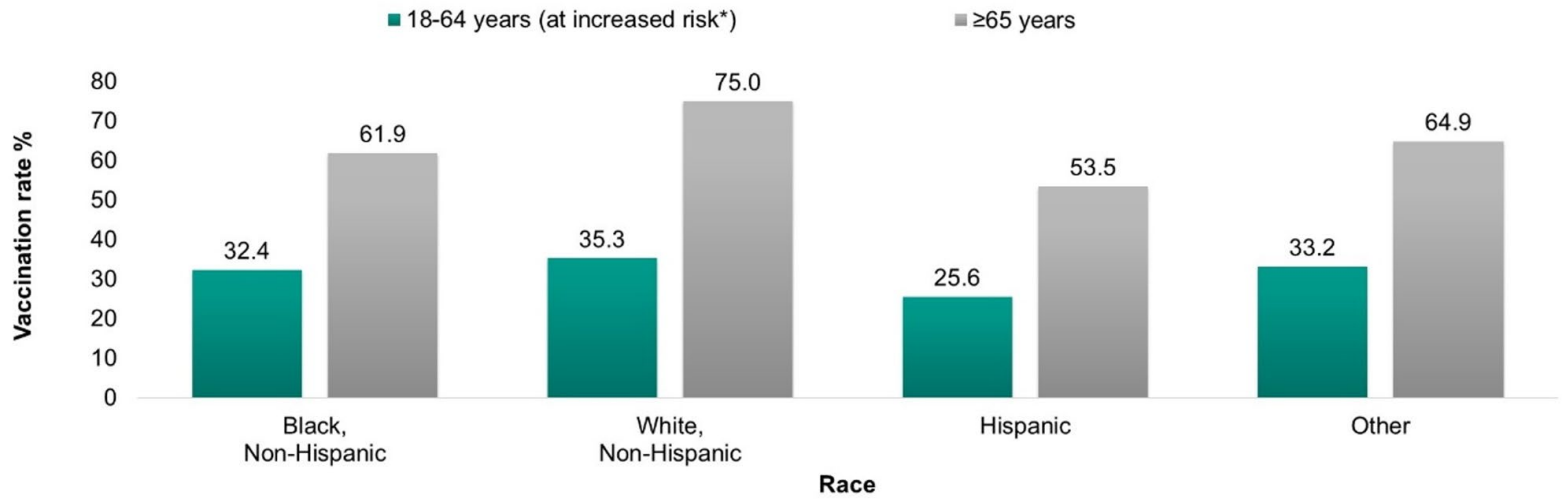
Pneumococcal vaccination rate by race, 2019. Source: CDC Adultvaxview data 2022 [14]. Pre-pandemic data presented. *Increased risk was defined as if the individual self-reported having current asthma, ever having diabetes, myocardial infarction, angina or coronary heart disease, being a current smoker, or ever having chronic obstructive pulmonary disease, emphysema, or chronic bronchitis, or cancer (excluding skin cancer), or ever had kidney diseases (excluding kidney stones, bladder infection or incontinence)

Additional disparities were evident in the rate of pneumococcal vaccination. In the US, pneumococcal vaccine coverage is higher among White adults compared to non-White adults. According to the CDC in 2019, the pneumococcal vaccination rate was highest among White adults compared to adults from other racial and ethnic groups, with Hispanic adults having the lowest vaccination rate (Fig. 2) [14].

### Disparities by urbanicity

Evidence demonstrated notable variation in pneumococcal disease burden by urbanicity in US adults, although data were limited.

#### Incidence

According to the CDC’s National Notifiable Diseases Surveillance System report from 2019, the incidence rate of IPD across the US was 8.0 per 100,000, with a wide range of variation across states (0.3 to 20.8 per 100,000 population in Virginia to Alaska, respectively) and regions (5.9 to 12.2 per 100,000 population in the South Atlantic to the Pacific regions, respectively) [15]. No studies on incidence by urbanicity were identified in our search.

#### Mortality

**Fig. 3:**
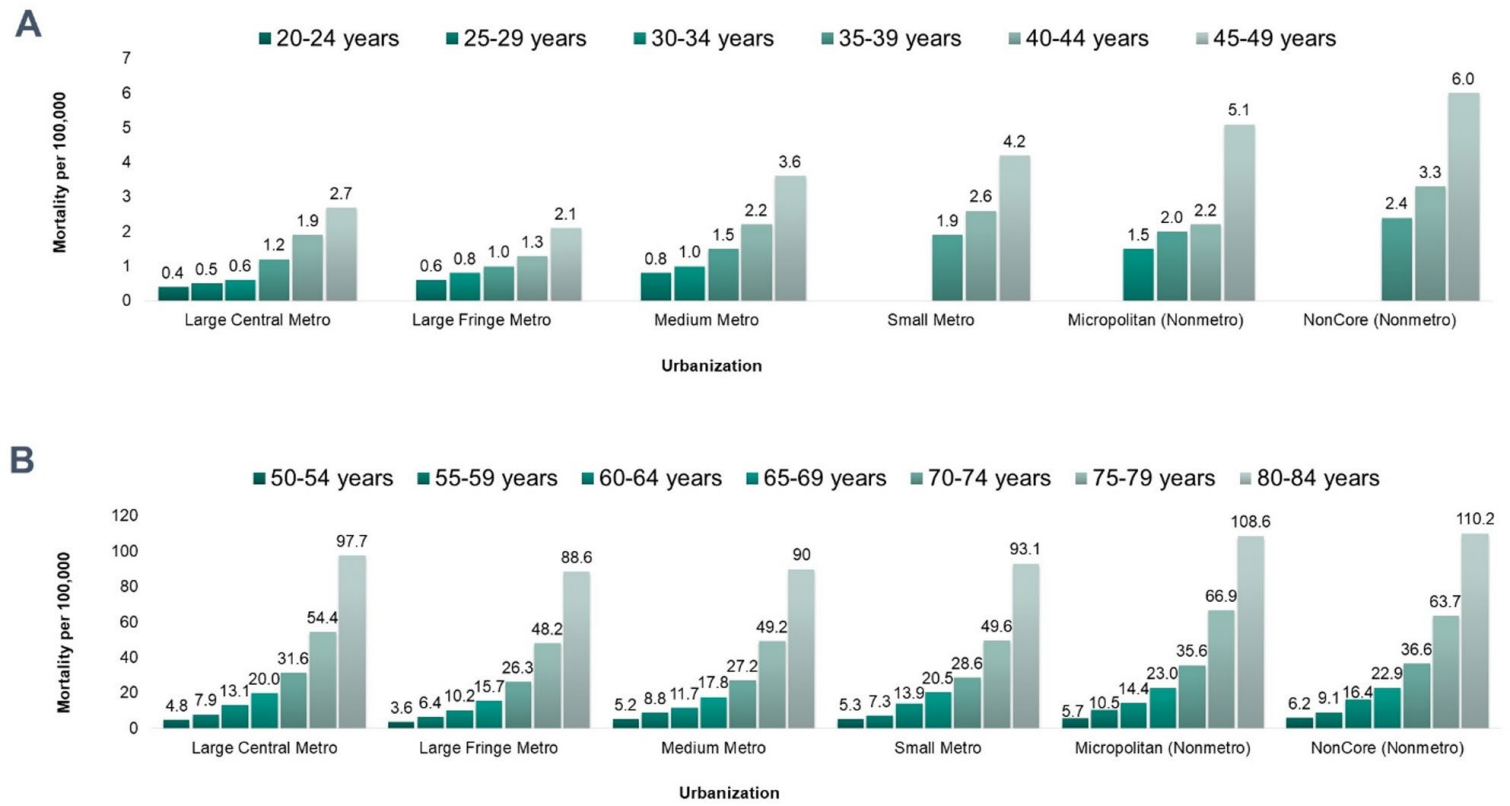
ACP* mortality by urbanicity and age based on CDC data, 2019. *ACP, all-cause pneumonia. Source: Adapted from CDC 2021 [13]. **A** presents ACP mortality by urbanization classification for age groups 20–24 years to 45–49 years. **B** presents ACP mortality by urbanization classification for age groups 50–54 years to 80–84 years. Pre-pandemic data is presented. Urbanization according to the 2013 NCHS Urban-Rural Classification Scheme for Counties: Large central metro: Counties in metropolitan statistical areas (MSAs) ≥ 1 million, contain or are part of the largest principal city, or have ≥ 250,000 residents. Large Fringe Metro: Counties in MSAs ≥ 1 million, but not classified as large central. Medium Metro: Counties in MSAs with populations between 250,000 and 999,999. Small Metro: Counties in MSAs with populations under 250,000. Micropolitan: Counties with urban clusters of 10,000–50,000 people, including nearby counties with commuting ties. Noncore: Counties not part of a micropolitan statistical area

Mortality data provided more detailed insights into urbanicity differences. In 2019, CDC WONDER database showed that among individuals aged ≥ 15 years, mortality due to pneumococcal pneumonia was higher in Noncore (most rural) and Small Metro areas (0.2 per 100,000) compared to other urban areas (0.1 per 100,000) [13]. ACP mortality rates were also higher in less urbanized areas and increased with age (Fig. 3). For younger adults (< 50 years), ACP mortality ranged from 0.4 to 2.7 per 100,000 in Large Central Metro areas and from 2.4 to 6.0 per 100,000 in Noncore areas. Among older adults (50–64 years), mortality ranged from 4.8 to 13.1 per 100,000 in Large Central Metro areas and from 6.2 to 16.4 per 100,000 in Noncore areas [13]. 

#### Healthcare resource utilization and costs

No recent US studies addressed healthcare resource utilization and costs by population density.

#### Vaccination rate

Vaccination patterns also revealed a rural–urban disparity, where adults living in rural or less urbanized regions have lower rates of pneumococcal vaccination. Between 2010 and 2021, a population-based study (*N* = 45,755) revealed that vaccination rates among Minnesota adults aged 19–64 years were lower in rural areas (36.3%) compared to urban areas (47.3%), highlighting reduced vaccination uptake in less urbanized regions. Additionally, the odds of vaccination were significantly lower outside of urban areas (OR, 0.77; 95% CI, 0.73–0.80; *p* < 0.001) [16]. In 2017, a cross-sectional analysis of administrative medical and prescription claims data across the US (*N* = 8,133,847) showed that older adults (≥ 65 years) in suburban/metropolitan areas had a significantly higher vaccination rate (85%) than those in urban (13.7%) and rural (1.3%) settings [17]. 

### Disparities by socioeconomic status

SES is an additional factor for which disparities were observed.

#### Incidence

In the US, adults of lower SES have a higher incidence, and higher odds of pneumococcal disease compared to adults of higher SES, although studies addressing disparities in pneumococcal disease incidence by SES are scarce.

A cross-sectional observational study (*N* = 268) conducted in the Southeastern US over the 2010–2019 period used area deprivation index (ADI) values (a scale of 1 to 100, where higher numbers indicate more disadvantages) to measure socioeconomic disadvantage. ADI measures the SES (based on education, income/employment, housing, and household characteristics) of a geographic area based on census data. Among adults < 65 years hospitalized with IPD, 41.7% were from more disadvantaged areas (ADI 79–100) and 27.2% were from less disadvantaged areas (ADI 2–79) [18]. Consistently, adult patients 18–64 years old in more disadvantaged areas had a higher risk of hospitalization for IPD compared to those from less disadvantaged areas (OR = 2.9, 95% CI 1.34–6.08, *p* < 0.05); however, there was no statistically significant difference in the odds of hospitalized IPD between disadvantaged and less disadvantaged groups in adults ≥ 65 years [18]. 

One retrospective study was identified within the timeframe of this review, reporting on disparities by employment status. Incidence data from the Truven Health Analytics MarketScan Commercial Claims and Encounters Database and the Truven Health Analytics Health and Productivity Management database in 2009 (*N* = 12,502,017) reported that early retirees and their adult dependents had a higher incidence of all-cause community acquired pneumonia (CAP) compared to active employees and their adult dependents (15.0 cases/100,000 versus 10.6 cases/100,000, respectively) [19]. 

#### Mortality

No recent studies addressed mortality by SES.

#### Healthcare resource utilization

Among the few published studies, income had a noticeable effect on healthcare resource utilization in US adults. In a retrospective study conducted in 2010–2014 in New York City (*N* = 4,614,108), hospitalization rates in adults ≥ 18 years old with all-cause CAP were observed to increase with higher levels of area-based poverty [20]. Adults in the ≥ 30 percentile of poverty had a hospitalization rate of 771.4 per 100,000 population per year compared to 303.7 per 100,000 for adults in the < 10 percentile of poverty [20]. 

#### Costs

Limited available data showed that employment status played a role in healthcare costs for US adults. In a retrospective, claims-based study conducted in 2009 (*N* = 12,502,017), the average annual healthcare expenses for active employees and their adult dependents were found to be 42% less than that for early retirees and their adult dependents ($20,961 versus $30,932, respectively), while there was no notable contrast in average cost per episode of all-cause CAP ($36,139 versus $32,133, respectively) [19]. 

#### Vaccination rate

The level of SES had a noticeable effect on vaccination rates in US adults. A population-based study (*N* = 45,755) conducted in southeastern Minnesota over the 2010–2021 period used the housing-based SES (HOUSES) Index tool to measure individual-level SES among 19–64 years old adults and found that SES predicted vaccination status (*p* = 0.038) [16]. Specifically, adults in the highest SES level were more likely to be fully vaccinated against pneumococcal disease than adults in the lowest SES (OR = 0.953, 95% CI 0.914–0.993, *p* = 0.022) [16]. 

Socioeconomic factors including income, education, employment status and home ownership were found to be associated with vaccination rates. In a 2017 analysis of administrative medical and prescription claims data (*N* = 8,133,847), the vaccination rates among US adults ≥ 65 years were the lowest among individuals in the lowest decile (10%) of annual household income compared to those in the highest decile (10%), showing a statistically significant difference (31% vs. 54%, respectively; *p* < 0.01) [17]. In 2017, adults ≥ 65 years who had low levels of education (less than high school and just a high school diploma or equivalent) showed lower uptake of PCV13 compared to those with a graduate or college degree (33%–42.3% vs. 48.6%–49.5%, respectively) [17]. In a population-based study (*N* = 45,755) conducted in southeastern Minnesota from 2010 to 2021, it was found that high-risk 19–64 years old adults who had a college degree were more likely to be vaccinated against pneumococcal disease than those who only had a high school education or less, although this was not found to be statistically significant (OR 1.014; 95% CI 0.981–1.048; *p* = 0.409) [16]. In 2017, adults ≥ 65 years who had blue-collar occupations had the lowest PCV13 vaccination rate compared to those who worked in offices or in sales (40.1% versus 44.7%, respectively) [17]. In 2017, adults ≥ 65 years who did not own a home had lower PCV13 vaccination rates than home owners in the same age group (33% vs. 45%, respectively) [17]. 

## Discussion

Available research have suggested that SDoH contribute to disparities in pneumococcal disease risk among US adults, either directly or indirectly, through factors such as occupation, living and working conditions, and comorbidities [11, 16, 18]. However, this topic remains underexplored, with existing research primarily relying on geography and race/ethnicity to characterize the impact of SDoH on pneumococcal disease outcomes [21–23]. The results of this TLR are consistent with available research and also highlight that race/ethnicity is most frequently identified as a factor associated with disparities in pneumococcal disease, suggesting that Black individuals face the highest pneumococcal disease incidence (increasing 2-fold when living in impoverished areas), mortality, hospital LOS, and healthcare costs compared to White individuals. Available data from the ABC surveillance system, which were recently presented at the CDC Advisory Committee on Immunization Practices (ACIP) meeting on October 23, 2024, showed that the incidence of IPD peaked at younger ages (55–59 years) in Black adults compared to non-Black adults (≥ 65 years) [24]. 

Our TLR also found that vaccination rates were highest among White individuals and lowest among Hispanic individuals; however, regardless of race and ethnicity, reported data suggests that additional effort is required to reach optimal vaccine coverage in the US adult population. Furthermore, adults residing in the most impoverished areas, with lower educational attainment, higher unemployment, and lacking home ownership or experiencing homelessness, experienced the highest pneumococcal disease burden and exhibited the lowest vaccination rates compared to those adults residing in areas with better SES. Deprivation increases the risk of infection and poor disease outcomes due to factors that facilitate transmission and reduce the ability to fight infections, such as overcrowding, poor access to healthy food and clean water, poor sanitation, and limited access to treatment and prevention.

The TLR results also demonstrate that data are lacking for certain PD outcomes. However, leveraging research on other prevalent vaccine-preventable diseases such as influenza or COVID-19 for which more data are available could be beneficial in understanding why disparities exist in pneumococcal disease. A recent study of the Kaiser Permanente Mid-Atlantic States (KPMAS) health system found an association between low influenza vaccination in Black adults and lack of registration on their online patient portal [25]. This patient portal contains access to their medical records, appointments and health education (including information on influenza vaccination), which could aid in the use of the health system and provide guidance on vaccination [25]. A study on COVID-19 vaccination found that the most significant challenges faced by unvaccinated racial and ethnic minorities included lack of access to transportation, inability to attend during vaccine availability hours, and difficulty figuring out how to make an appointment [26]. Additionally, geographical barriers such as long travel distances and a limited number of vaccination sites in low-income neighborhoods with a high proportion of Latino and Black residents contribute to COVID-19 vaccine inaccessibility [27]. Overall, these findings represent common challenges that unvaccinated racial and ethnic minorities face in the US.

Recent data also shows that non-White adults are less likely to be diagnosed with chronic medical conditions associated with an increased risk of pneumococcal disease, despite having laboratory scores above the threshold for diagnosis [28]. Additionally, non-White adults continue to have lower rates of screening and therefore lower rates of awareness, treatment, and control of their chronic diseases compared to non-Hispanic White individuals, despite the implementation of screening recommendations for all, putting them at high risk of pneumococcal infection and poor disease outcomes [28]. The use of self-reporting measures and beliefs that some minorities, including Asians, have lower prevalence of common medical conditions may adversely affect screening practices within these groups and potentially worsen disparities [28]. SDoH, such as whether an individual is foreign-born or US-born, years lived in the US, and English proficiency, highlight cultural differences contributing to gaps in cancer screening, for example, where foreign-born Asian Americans often view screening as a response to symptoms rather than a preventive measure [29]. 

Recognizing that social determinants of health significantly influence health outcomes and access to healthcare, it is crucial to incorporate these factors into research. This approach can deepen our understanding of the drivers of health disparities, guiding the development of more effective, targeted interventions to address them.

The recent ACIP decision (October 2024) to lower age-based recommendations for pneumococcal vaccination of adults starting at age 50 years could play an important role in addressing health disparities by including more of these vulnerable adults [30]. Further research is needed to assess the impact of this updated recommendation. Additionally, gaining a deeper understanding of the barriers to vaccination and association with characteristics for which disparities were observed is essential for designing solutions to overcome these challenges.

This review included all-cause pneumonia in the search strategy due to possible underreporting of pneumococcal pneumonia. Reviewed literature lacked recent studies on pneumococcal pneumonia specifically which include data on the incidence by race, ethnicity, urbanicity, socioeconomic status and similar data on mortality as well as data on the healthcare resource utilization and costs overall. Therefore, results reported in this review include pneumonia caused by any pathogen unless otherwise stated. Other gaps in the literature include lack of evidence on the overall mortality rates by SES, healthcare utilization and costs by population density, and the incidence of pneumococcal disease by urbanicity. These gaps hinder a comprehensive understanding of how socioeconomic and geographic factors affect the clinical and economic burden of pneumococcal disease, highlighting the need for further research.

## Conclusion

There are significant disparities in the burden of pneumococcal disease among US adults with higher disease burden and lower pneumococcal vaccination rates among Black individuals and adults living in rural areas, with lower education and lower income. There is paucity of studies examining the role of SDoH in pneumococcal disease outcomes, and additional studies are needed to examine further disparities in pneumococcal disease according to race, geography, urbanicity, income, education, and employment.

The results of this study aid in the identification of adult populations in the US who may benefit from pneumococcal disease prevention. The recent ACIP decision to lower age-based recommendation for pneumococcal vaccination of adults to age 50 years may help address disparities. Additional targeted interventions to increase VCR among racial/ethnic groups and individuals living in rural areas, those with lower education and lower income may help address the higher pneumococcal disease burden in these vulnerable populations.

## Data Availability

No datasets were generated or analysed during the current study.

## Abbreviations

ABCs: Active Bacterial Core Surveillance
ACP: All-cause pneumonia
ACIP: Advisory Committee on Immunization Practices
CAP: Community-acquired pneumonia
CDC: Centers for Disease Control and Prevention
HIV: Human immunodeficiency virus
IPD: Invasive pneumococcal disease
LOS: Length of stay
NBPP: Non-bacteremic pneumococcal pneumonia
SDoH: Social determinants of health
SES: Socioeconomic status
TLR: Targeted literature review
